# Efficacy of manual therapy by different healthcare professionals on pain and function in temporomandibular disorders: a systematic review of randomized controlled trials

**DOI:** 10.1080/10669817.2026.2629282

**Published:** 2026-02-28

**Authors:** Rita Chiaramonte, Martina Ferrillo, Alessandro de Sire, Michele Vecchio

**Affiliations:** a Physical Medicine and Rehabilitation Unit, AOU Policlinico G. Rodolico, University of Catania, Catania, Italy; b Dentistry Unit, Department of Health Sciences, University of Catanzaro “Magna Graecia”, Catanzaro, Italy; c Physical Medicine and Rehabilitation Unit, Department of Medical and Surgical Sciences, University of Catanzaro “Magna Graecia”, Catanzaro, Italy; d Research Center on Musculoskeletal Health, MusculoSkeletalHealth@UMG, University of Catanzaro “Magna Graecia”, Catanzaro, Italy; e Department of Biomedical and Biotechnological Sciences, University of Catania, Catania, Italy

**Keywords:** Temporomandibular joint disorders, temporomandibular disorders, musculoskeletal manipulations, manual therapy, oral rehabilitation, rehabilitation

## Abstract

**Background:**

Manual therapy is widely used in temporomandibular joint disorders (TMD), but its effectiveness across different professional practices and techniques remains unclear. This systematic review of randomized controlled trials (RCTs) aims to evaluate the efficacy of manual therapy in alleviating pain and improving functional recovery in patients with different TMD subtypes, considering the roles of various healthcare professionals.

**Methods:**

A systematic search was conducted across PubMed, Scopus, Cochrane and Web of Science to identify RCTs presenting participants with a diagnosis of TMD, manual therapies as interventions; other therapeutic modalities, placebo, or sham therapy as comparisons; pain intensity and maximum mouth opening (MMO) as outcomes.

**Results:**

From 561 initial records, 18 RCTs met the inclusion criteria and were included in the analysis. Overall, manual therapy was associated with reductions in pain and improvements in MMO across different professional background and techniques. However, the certainty of evidence was limited, and effects were predominantly observed in the short term. Physiotherapists in rehabilitation settings showed the most consistent outcomes, particularly documented in Brazil. Chiropractic approaches were common in Australia, USA, and England, while Japan focused on intra-oral manipulations by dental and maxillofacial specialists. Italy, Spain, Greece and Turkey stand out for their interdisciplinary approach independently by chiropractors, physiotherapists, or osteopaths in TMD management.

**Conclusions:**

This systematic review suggests that manual therapy may be associated with short-term improvements. Variability in techniques, professional settings, and study designs precludes firm conclusions regarding long-term effectiveness or superiority of specific approaches. Physiotherapy manipulation emerged as the most used technique. Further research should explore long-term outcomes and multiprofessional collaboration.

## 1. Introduction

Temporomandibular disorders (TMD) are a group of clinical conditions involving the masticatory muscles, the temporomandibular joint (TMJ), and associated structures [1]. The prevalence of TMD varies according to age, sex, geographic region and specific diagnostic subtype [1]. Overall, TMD affect approximately 31% of adults and elderly, and 11% of children and adolescents [1,2]. Prevalence is notably high in South America (47%), Asia (33%) and Europe (29%) [1].

According to the *Diagnostic Criteria for Temporomandibular Disorders* (DC/TMD), common subtypes include muscle disorders (e.g. myalgia, with or without referral), disc displacement (with or without reduction, limited opening), and joint-related conditions such as arthralgia or degenerative changes [3]. Despite this classification system, the complex and multifactorial nature of TMD, together with overlapping clinical presentations often complicates differential diagnosis [4]. In routine practice, the absence of standardized diagnostic pathways and universally accepted treatment protocols frequently results in empirical management, particularly in the early stages of care [5–10]. In this context, the *International Network for Orofacial Pain and Related Disorders Methodology group of the International Association for Dental, Oral and Craniofacial Research* (INfORM/IADR) recommends conservative, reversible approaches aimed at pain reduction and functional improvement [11]. Within this framework, manual therapy has emerged as a promising option. It encompasses various techniques, including high-velocity low-amplitude manipulation (HVLA), soft tissue manipulation and traction applied to the TMJ or spine [12]. These hands-on approaches are highly operator-dependent and require advanced clinical training to ensure safety and efficacy [13]. Outcomes are influenced by the practitioner’s expertise, precision, and ability to tailor techniques to the patient’s specific clinical presentation [13].

Manual therapy may be administered by different professionals, including physiotherapists, osteopaths, chiropractors, dental specialists and medical doctors from various specializations [14]. This diversity contributes to heterogeneity in the literature, as treatment outcomes are shaped by differences in clinical approach [15,16], underlying etiology, and involvement of structures such as joints (TMJ or spine), muscles, and neuropathic components – factors that complicate differential diagnosis [17,18].

The primary aim of this systematic review of randomized controlled trials (RCTs) was to identify which manual therapy techniques yield the most clinically relevant improvements in TMD management. Specifically, the review assessed the effectiveness of manual therapy compared to other interventions – such as splint therapy, pharmacological treatments, sham or placebo procedures, and no treatment – in reducing pain and improving functional outcomes, particularly maximum mouth opening (MMO). Effectiveness was assessed using patient-reported pain intensity measures and objective functional evaluations. Secondary aims included: 1) Does the effectiveness of manual therapy vary across different professional practices? 2) Are certain TMD subtypes (e.g. myogenic, disc displacement, myofascial) more responsive to manual therapy? 3) Which professional groups, based on author affiliations and nationality, are the most involved, in TMD treatment as reported in clinical studies?

## 2. Methods

This systematic review was conducted in accordance with the *Preferred Reporting Items for Systemic Reviews and Meta-analyses* (PRISMA) statement [19], and structured using the *Population, Intervention, Comparison, Outcome, Study design* (PICOS) guidelines [20].

The review protocol was registered in *the International Prospective Register of Systematic Reviews* (PROSPERO) (CRD42024579074).

### 2.1. Search strategy and data extraction

**Figure 1.**
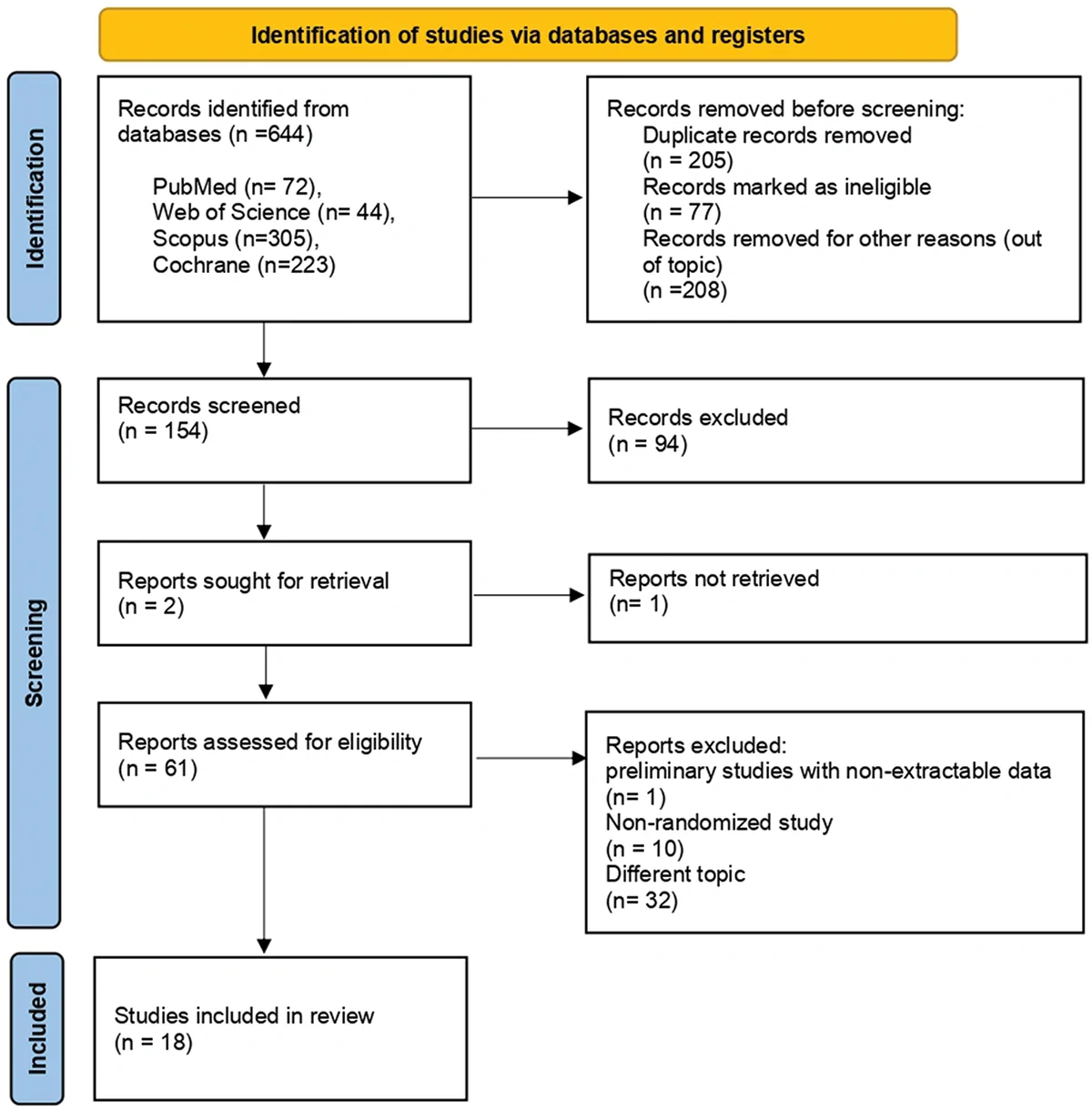
PRISMA 2020 flow diagram.

A comprehensive literature search was performed across the following databases: PubMed, Scopus, Cochrane Library, and Web of Science (WOS), covering the period from database inception to March 2025, (Figure 1). For PubMed, the search strategy was: (Manipula* OR ‘Manual Therapy’[tiab] OR ‘Spinal Manipulation’[tiab] OR ‘Chiropractic Manipulation’[tiab] OR ‘Osteopathic Manipulation’ [tiab] OR ‘Musculoskeletal Manipulations’[tiab] OR ‘Orthopaedic Manipulations’[tiab]) AND (‘Temporomandibular Joint Disorders’[MeSH] OR Temporomandibul* [tiab] OR TMJ[tiab] OR TMD[tiab]). For Scopus, WOS, and Cochrane Library, the strategy was adapted as: (Manipula* OR ‘Manual Therapy’ OR ‘Spinal Manipulation’ OR ‘Chiropractic Manipulation’ OR ‘Osteopathic Manipulation’ OR ‘Musculoskeletal Manipulations’ OR ‘Orthopaedic Manipulations’) AND (Temporomandibul* OR TMJ OR TMD OR ‘Temporomandibular Joint Disorders’). Filters were applied to include clinical studies, published in English, with the subject area restricted to *Medicine* in Scopus. Additionally, reference lists of relevant systematic reviews were manually screened to identify further eligible studies.

Two authors independently screened titles and abstracts, followed by full texts assessment of potentially eligible articles. Discrepancies were resolved through discussion or, if necessary, by consulting a third authors. To ensure consistency, the study selection and data extraction process were repeated independently by one author during the final update phase.

The extracted data included the study characteristics (authors, year of publication, study design), samples characteristics (age, TMD diagnosis), intervention details (type of manual therapy, anatomical structures treated, timing of interventions), follow-up duration, and outcome measures.

### 2.2. Eligibility criteria and study selection

Eligibility was defined according to the PICOS framework [20]. For *population* (P), the studies included adults with a clinical diagnosis of TMD. Manual therapy *intervention* (I), including HVLA, myofascial release, spinal manipulations and other hands-on approaches, regardless of the provider’s professional background (e.g. physiotherapists, chiropractors, osteopaths, dental specialists and medical doctors); as *comparators* (C), alternative treatments such as splints, behavioral therapies, pharmacological interventions, sham treatment or no-treatment. As primary *outcomes* (O), the analysis focused on results on pain and MMO after treatments that showed benefits in the examined studies [21–26] (Table 1). *Study design* (S) included RCTs.

**Table 1.** Main characteristics of the randomized controlled trials included in the present systematic review. The included RCTs were highly heterogeneous in terms of populations, interventions, targeted structures, treatment dosage, and follow-up. Most studies reported short-term improvements in pain and/or maximal mouth opening, while evidence for long-term effects was limited. Differences across techniques and professional categories should be interpreted descriptively, as they likely reflect study design and intervention variability rather than causal superiority.

First Author, year of publication	Patients characteristics	TMD diagnosis intervention	Manual therapy	Targeted structures	Treatment session	Follow up	Outcomes	Conclusions
Bortolazzo, 2015 [27]	Group 1: 5 pt received upper cervical manipulation. Group 2: 5 pt underwent maneuvers without therapeutic effects.	EMG	Upper cervical manipulation at occipital, atlas and axis. Techniques included right and left rotation manipulations with a slight upward pull, followed by a high-speed, short-amplitude pulse.	Masticatory and temporal muscles	Once a week	Assessments after 1st intervention and 48 hours after the 5th intervention.	Significant increase (*p* < 0.05) and effect during mouth opening in group 1	The upper cervical manipulation demonstrated a balance of peak value on EMG of masticatory muscles and an increase of the MMO in women with myogenic TMD
Capellini, 2006 [28]	Group 1: 6 pt 20.3 ± 1.2 y (massage + EMG); Group 2: 6 pt 20.5 ± 1.4 y (EMG only)	VAS, EMG	Superficial and deep sliding, friction, compressive stationary, kneading, kneading with distension, rolling and superficial sliding techniques	Masseter and anterior temporalis muscles	15 massage-sessions daily, of 30 min. for 3 weeks. 4 EMG-sessions (at T0, 1st, 15th and 30th days)	After treatment	RMS-MRP of the right masseter in the experimental group at 1st EMG session was higher than at 2nd EMG session (*p* = 0.03). The differences between the remaining muscles of the experimental group were not statistically significant	The results allowed to state that (1) RMS of right masseter in experimental group at the 1st EMG session was higher than at the 2nd EMG session and that (2) a statistically significant reduction was found for VAS values after massage session
Corum, 2018 [21]	Group 1: 18 pt received cervical spinal manipulation + neck exercise Group 2: 19 pt received sham manipulation + neck exercise Group 3: 18 pt received patient education only 27.0 ± 6.3 y	NRS, PPT, MMO	Manipulation hand: deep contact with deep dorsal to ventral force. Sham spine manipulation was performed at different spinal segment without therapeutic intent.	Latissimus dorsi, pectoralis major muscle	Home exercise program three days per week, with one rest day between session. Three sets of 5 repetitions per session, with a 30–60 second rest between sets. Exercises included cervical range-of-motion warm-ups, stretching, and strengthening for the neck and upper body.	1-month follow-up	In terms of pain, significant differences were observed in the cervical spinal manipulation + neck exercise	HVLA of the upper cervical spine + neck exercise can be effective on pain and dysfunction in patients with chronic TMJ
Cuccia, 2010 [24]	Group 1: 25 pt received osteopathic manual therapy 40.6 ± 11.03 y Group 2: 25 pt received conservative therapy 38.4 ± 15.33	VAS, MMO and lateral range of motion	Conservative treatment: gentle muscle stretching and relaxing exercises. Manual therapy: myofascial release, balanced membranous tension, muscle energy techniques, myofascial release, joint articulation, HVLA and cranial-sacral therapy	Masticatory muscles	15–25 min for 2 weeks	6 months	Both groups improved significantly over six months. Group 1 required significantly less medication (*p* < 0.001).	The two therapeutic modalities had similar clinical results in group 1, even if the use of medication was greater in group 2.
DeVocht, 2013 [29]	80 pt Group 1: 20 pt received reversible interocclusal splint therapy (RIST) Group 2: 6 pt received *Activator Method Chiropractic Technique* Group 3: 19 pt sham *Method Chiropractic Technique* Group 4: 21 pt practiced self-care	NRS, Oral Health Impact Profile, Bothersomeness index	Each specific manipulation – called ‘adjustment’ – is given with a hand-held, spring-loaded instrument. The AMCT A spring-loaded hand-held instrument delivers a quick, shallow thrust targeting the spine, extremities, and temporomandibular joint.	–	–	2 and 6 months	Adjusted mean change in pain over six months, (11-point numerical rating scale): RIST 2.0 (95 % CI: 1.1–3.0), self-care 1.7 (0.9- 2.5), *Activator Method Chiropractic Technique* 1.5 (0.7–2.4), sham 1.6 (0.7–2.5)	The study recommends a full-scale to evaluate *Activator Method Chiropractic* effectiveness for chronic myofascial TMD.
Dunning, 2022 [25]	120 pt Group 1: 62 pt 40.2 ± 12.4 y Control Group 2: 58 pt 43.0 ± 13.1 y	VAS, GROC, MMO	Group 1: needling + upper cervical spinal manipulation, HVLA Control group 2: interocclusal splint therapy, diclofenac, and 10 mins of non-thrust joint mobilization	Lateral pterygoid, superficial masseter, temporalis muscle, and peri-articular capsule of the posterior TMJ	Eight sessions of dry needle, 1–2 times per week for 20 min over 4 weeks	2 and 6 weeks, 3 months	Group 1 showed significantly greater reductions in jaw pain (*p* < 0.001) and active pain-free mouth opening (*p* < 0.001) compared to Group 2 at the 3-month follow up.	Dry needling and upper cervical spinal manipulation were more effective than splint therapy, diclofenac, and TMJ mobilization
Gębska, 2023 [30]	Group 1: 82 pt Group 2: 104 healthy individuals with no treatment	sEMG of the masseter muscles at baseline and during exercise	Group 1, subgroup: a) therapeutic exercises and manual therapy b) massage and therapeutic exercises, manual therapy c) post-isometric muscle relaxation and therapeutic exercises	Masseter muscle	Over a period of 10 days (excluding weekends).	After the 5th and 10th therapeutic day	After day 6 of therapy, the groups obtained a significant difference (*p* = 0.0001), especially in group 2	Massage showed better analgesic effects than post-isometric muscle relaxation. Manual therapy and therapeutic exercises are safe, effective, and simple interventions for myogenic TMD.
Gesslbauer, 2018 [31]	40 pt Group 1: 20 female pt received osteopathic manipulation Group 2: 20 pt received osteopathy in the cranial field	VAS, Helkimo Index, SF-36 Health Survey.	Techniques included: articulatory techniques, balanced ligamentous tension and articular strain, cranial osteopathy, counter strain, facilitated positional release, HVLA, integrated myofascial and neuromusculoskeletal release, muscle energy, soft-tissue technique, visceral manipulative treatment.	Masticatory muscles	Five treatments of 30 min. weekly	At the end of the treatment	Patients in both groups showed significant reduction in VAS (*p* = 0.001), Helkimo Index (*p* = 0.02; *p* = 0.003), SF-36 subscale ‘Bodily Pain’ (*p* = 0.04; *p* = 0.007) after five treatments	Osteopathic manipulative treatment and osteopathy in the cranial field reduced pain, and improved temporomandibular joint, with positive impact on quality of life
Gomes, 2014 [32]	42 pt 1) Massage therapy group14 pt 30.10 ± 5.80 2) Occlusal splint group: 14 pt 29.70 ± 3.10 3) Healthy group 14 s 30.87 ± 6.20	MMO	Sliding and kneading maneuvers.	Masseter and temporal muscles	3 weekly 30 minutes sessions for 4 weeks	Before and after treatment	Significant increases in ROM were found for all measures in both the massage and occlusal splint groups (*p* < 0.05).	Massage therapy on the masticatory muscles and the use of an occlusal splint led to an increase in mandibular ROM similar to that of the asymptomatic comparison group with regard to MMO
Guarda-Nardini, 2012 [33]	30 pt Group A single-session botulinum toxin injections Group B: multiple-session Fascial Manipulation	VAS, MMO	Single session of multiple botulin toxin injections (150 U) into temporalis and masseter muscles.	Temporalis and masseter muscles	Three (±1) 50 min sessions of Fascial Manipulation weekly, totaling 150 (±50) min over 2–4 weeks	Baseline, at the end of treatment and at a three-month follow-up.	Group A: Reduced pain from baseline to 5.2 post-injection and 4.8 at the 3-month follow-up and slight increase in MMO 51.4 mm; left latero 8.7 mm; protrusion 7.0 mm; right latero 8.7 mm), Group B: Reduced pain to 2.1 at the end of treatment and 2.5 at the 3-month follow-up. MMO revealed stable values with minimal variance	Fascial Manipulation was slightly superior to reduce subjective pain perception, and botulinum toxin injections being slightly superior to increase jaw range of motion.
Kalamir, 2012 [34]	93 pt Group 1 IMT 31 pt manipulation, 35 ± 6.7 Group 2 IMTESC 31 pt IMT + education and ‘self-care’ exercises 34 ± 6.1 Group 3 31 pt on a wait-list, 35 ± 5.0	Pain, MMO, GROC	Intraoral temporalis release Intraoral medial and lateral pterygoid technique Intraoral sphenopalatine ganglion technique	Temporalis, pterygoid, masseter muscle	Two treatment sessions per week of 10–15 minutes for 5 weeks	6 months, 1 year	Statistically significant improvement at rest, opening and clenching pain, opening scores, and global reporting of change (*p* < 0.05) in both treatment groups compared with the controls at 6 months and 1 year	Chiropractic IMT and IMTESC demonstrated superior efficacy for chronic myogenous TMD over the course of 1 year, with IMTESC showing additional benefits.
Kalamir, 2013 [35]	46 pt Group 1: intra-oral myofascial therapies Group 2: education, self-care and exercise	NRS, MMO	Group 1 1) Intra-oral temporalis release 2) Intra-oral medial and lateral pterygoid (origin) Technique 3) Intra-oral sphenopalatine ganglion technique Group 2: 1) Guided and controlled jaw excursions 2) Post – isometric stretches	Temporalis muscle, medial pterygoid muscle, masticatory muscles	Two sessions per week for five weeks	6 weeks	Group 1 showed significantly lower average pain for all primary outcomes at the 6-week follow-up compared to the ESC group (*p* < 0.001).	Intraoral myofascial therapies demonstrated greater short-term effectiveness (6 weeks) compared to education, self-care, and exercise.
Nagata, 2018 [36]	61 pt Group1: 30 pt conventional treatment 48.2 ± 21.1 Group 2: 31 pt conventional treatment + manipulation 50.7 ± 18.3	NRS, MMO	Jog Manipulation: A novel technique combining four steps performed with a gauze pivot placed on the last molar: 1. Closing with fulcrums on both sides; 2. Side-to-side motion, 3. Opening motion, 4. Closing with fulcrum on the impaired side.	Jaw and associated musculoskeletal structures	10 repetitions, three or five times per day	18 weeks	No statistical difference was observed between the groups except for mouth opening limitation after first visit – mean average increment 6.86 mm (95% CI = 8.12–5.55)-	The efficacy of manipulation appears limited in the long term. Enhanced therapeutic exercises showed effects similar to those of manipulation over prolonged observation.
Oliveira-Campelo, 2010 [26]	Group 1: Atlanto-occipital joint thrust Group 2: inhibition technique applied to suboccipital muscles Group 3: control group, no intervention.	Pressure pain thresholds over latent trigger points in the masseter and temporalis muscles, MMO	A slight traction is cranially introduced and a high-velocity low-amplitude thrust is performed in the direction of traction with a gentle left rotation force. Suboccipital muscle inhibition technique:	Atlanto-occipital Joint thrust and Suboccipital Muscle Inhibition Technique	One treatment session	Immediate after treatment	Significant changes in pressure pain thresholds over masseter (*p* < 0.01) and temporalis (*p* = 0.003) muscle latent. Improvement in MMO (*p* < 0.001) in manipulative and soft tissue groups.	Both the atlanto-occipital manipulation and the soft tissue technique targeting suboccipital muscles resulted in immediate increases in pressure pain thresholds over latent TrPs and MMO
Packer, 2014 [37]	Experimental group (n. 16), submitted to upper thoracic manipulation, 23.5 (21–25) Placebo group (n. 16), submitted to sham procedures 26 (22–29)	VAS	The upper thoracic manipulation (high-velocity, low-amplitude thrust) was applied to the T1 vertebral segment	Masticatory and temporal muscles	One treatment	Immediately after the procedure as well as after 48–72 hrs	No significant group-by-time interaction was found (*p* > 0.05)	Upper thoracic spinal manipulation does not lead to a reduction in pain in women with temporomandibular disorder.
Reynolds, 2020 [23]	Group 1: 25 pt spinal thrust Group 2: 25 pt sham manipulations	NRS, MMO, Tampa Scale of Kinesio-phobia, Jaw Functional Limitation Scale, GROC	Suboccipital release for 2 min., Education and review of a home exercise program. HVLA	–	The mean numbers of treatment were: 4.17	One and four weeks	Significant interactions were noted in JFLS (*p* = 0.60) and TSK-TMD (*p* = 0.80).	Significant differences between groups were noted for Tampa Scale of Kinesio-phobia, Jaw Functional Limitation Scale and GROC.
Tuncer, 2013 [22]	Group 1: 20 pt home physical therapy 34.8 ± 12.4 Group 2: 20 pt manual therapy + home physical therapy 37.0 ± 14.6	VAS, MMO	Home physical therapy: ergonomic advice, breathing exercises, relaxation techniques, posture correction exercises and mandibular exercises such as active and repetitive assisted muscle stretching, mouth opening and closing, medial and lateral gliding and resistance exercises	Soft tissue mobilization (intra- and extra-oral deep friction massage of painful muscles), TMJ mobilization (caudal and ventro caudal traction, ventral and mediolateral translation), TMJ stabilization (gentle isometric tension exercises against resistance), coordination exercises (guided opening and closing jaw movements), cervical spine mobilization (traction and translation), post isometric relaxation and stretching techniques for masticatory and neck muscle	30 minutes three times a week for four weeks.	Four weeks	A four-week period of manual therapy + home physical therapy has a clinically significant effect on pain and MMO (*p* < 0.001)	In the short term, manual therapy in conjunction with home physical therapy is more effective than home physical therapy alone for the treatment of TMD
Yoshida, 2005 [38]	305 pt Group A: 204 pt Group B: 101 pt 18 to 74 years	VAS, MMO	Group A: jaw manipulation and non-steroidal anti-inflammatory drug therapy Group B: no specific treatment	Musculoskeletal manipulations	–	–	Success rates varied by age and timing of treatment: 18–19 years age group: 100% success rate for treatment given on the day of diagnosis. 20–49 years age group: 87.5% success rate for manipulation. 50–59 years age group: 78.2% success rate for manipulation. Timing: Treatment within 1–2 days of onset achieved a 100% success rate.	Simple jaw manipulation is highly effective for closed lock symptoms when applied early, particularly in younger patients.

Abbreviations: Temporomandibular joint TMJ; Temporomandibular disorders TMD; Electromyography EMG; Patient pt; Minutes min; Numerical Rating Scales NRS; Coping Strategies Questionnaire 27 CSQ-27; EuroQol EQ-5D-5 L; Hospital Anxiety and Depression Scales HADS; Visual Analogue Scale VAS; Central Sensitisation Inventory CS; Graded Chronic Pain Scale GCPS; Maximal Mouth Opening MMO; Randomized Controlled Trial RCT; Root Mean Square RMS; Manual Therapy part of Rehabilitation Program of Medical Doctors Ph; Global Rating of Change GROC; Pressure Pain Thresholds PPT; high-velocity low-amplitude manipulation HVLA; Intraoral temporalis release IMT; Intraoral sphenopalatine ganglion technique IMTESC.

Studies were included regardless of the provider’s profession, country of origin, or timing of intervention. Manual therapy was found to be effective in reducing pain immediately after treatment, independent of the professional category (Table 2). This effect was consistent across studies involving physiotherapists and other healthcare professionals [21–25].

**Table 2.** GRADE quality of evidence of the studies.

Quality assessment					Summary of findings	Quality of evidence GRADE
N° of studies	Limitations	Inconsistency	Indirectness	Publication bias	Characteristics of	
18 studies	No blinded RCTs: Corum 2018 [21], Guarda-Nardini 2012 [33]	No serious inconsistency	No serious indirectness	Unlikely	Population: Adults Intervention: Manual therapy Comparison: Control (no therapy, different treatment than manual therapy) Outcomes: Improvement in pain and function	Moderate-high

Eligible studies were conducted in clinical settings where manual therapy is routinely administered, such as outpatient clinics, rehabilitation centers, physiotherapy practices, chiropractic offices, and osteopathic clinics. No restrictions were applied regarding the year of publication.

Exclusion criteria were: (1) non-RCTs (e.g. observational studies, case series, case control studies, case reports, editorials, expert opinions, interviews); 2) studies not published in English; 3) insufficient or inadequately reported outcome data; (4) unclear description of manual therapy techniques, or studies focusing on non-manual interventions (e.g. pharmacological treatments, surgical procedures, behavioral therapies, physical therapy, acupuncture), or where manual therapy was not the primary intervention; 5) participants with significant comorbidities affecting the head, neck, or jaw (e.g. rheumatoid arthritis, fibromyalgia, non-TMD headaches); 6) gray literature and unpublished data.

### 2.3. Manual therapy: definition and scope

In this review, *manual therapy* was defined as a broad category of hands-on interventions applied to the temporomandibular region and related musculoskeletal structures. This definition included high-velocity low-amplitude (HVLA) manipulations, myofascial release, fascial manipulation, intra-oral techniques, massage, and other joint or soft-tissue manual approaches. This inclusive definition was adopted to reflect both real-world clinical practice and the terminology used in the existing literature, where these interventions are frequently grouped under the umbrella term of manual therapy. However, it is acknowledged that these techniques differ substantially in their proposed mechanisms of action, therapeutic intent and anatomical targets (Table 3). For this reason, while manual therapy was evaluated as a global intervention to address the primary aim of the review, technique-specific findings were analyzed descriptively and interpreted with caution. Direct comparisons between different manual therapy techniques were not intended to imply equivalence or superiority, but rather to illustrate patterns of effectiveness reported across heterogeneous interventions.

**Table 3.** Comprehensive overview of TMD diagnoses and treatment.

Type of intervention	Primary target	Rationale for grouping	Description of intervention		Professionals performing manual therapy	Affiliations of the authors	Countries	References
Fascial manipulations	Deep fascia of masticatory muscles	Techniques primarily aimed at restoring fascial sliding and reducing densification, distinct from muscle-focused soft-tissue approaches.	Myogenic temporo-mandibular joint treatment with localized pressure and specific movement sequences		Physiotherapist	Department of Physiotherapy	University of São Paulo, Brazil.	Capellini 2006 [28]
						− Department of Rehabilitation Muscuoskeletal System − Department of Dental Prosthetics	University in Szczecin, Poland.	Gębska 2023 [30]
						Department of Maxillofacial Surgery	University of Padova, Italy	Guarda-Nardini 2012 [33]
Myofascial Release	Masseter, temporalis, pterygoid and cervical muscles	Intervntions focusing on muscle – fascia units and trigger point modulation.	Trigger-point releases with sustained manual pressure and stretching applied to superficial and deep myofascial tissues, intra- and extra-orally.		Dental nurse	Department of Chiropractic	Macquarie and Brisbane University, Sydney Australia, Bournemouth University, England	Kalamir 2012 [34]
					Chiropractor	− Department of Chiropractic − School of Exercise Science	Macquarie University, Sydney Australia	Kalamir 2013 [35]
					Physical therapist	Department of Physiotherapy and Rehabilitation	University, Ankara, Turkey. Harvard University, Boston, USA	Tuncer 2013 [22]
Intra-Oral Manipulations	Medial and lateral pterygoid, temporalis, TMJ capsule.	Techniques requiring intra-oral access with direct mechanical action on deep structures.	Treatment of closed lock by manipulation: Thumb pressure applied against the labial side of upper anterior tooth while the lingual side of the lower incisor was pulled with the forefinger		–	Department of Oral and Maxillofacial Surgery	Wakayama and Osaka Dental University, Japan	Yoshida 2005 [38]
**Upper Cervical spine High-Velocity Low-Amplitude Manipulation**	C1–C7 joints and suboccipital region.	Spinal manipulative interventions hypothesized to influence TMD via neurophysiological mechanisms.	High-velocity technique targeting upper cervical spine – Atlanto-Occipital Joint Thrust		Physical therapist specialized in Osteopathy	− Program in Oral and Dental Biology − Program in Rehabilitation − Program in Human Movement Sciences	Universidade de Piracicaba and São Paulo, Brazil.	Bortolazzo 2015 [27]
					Medical Doctors	Department of Physical Medicine and Rehabilitation	Istanbul University, Turkey	Corum 2018 [21]
					Physiotherapist	− Department of Physical Therapy − Osteopractice Physical Therapy	1. Universidad Alcorcón, Spain, 2. University of Thessaloniki, Greece	Dunning 2022 [25]
					–	− Osteopathic School − Department of Physical Therapy	1. Universidad de Sao Carlos, Brasil. 2. Universidad de Salamanca and Madrid Spain	Oliveira-Campelo 2010 [26]
					Clinicians of the American Academy of Orthopaedic Manual Physical Therapists	Department of Physical Therapy and Health Science	University of Peoria, Waco, Lauderdale, Manchester, USA	Reynolds 2020 [23]
**High-Velocity Low-Amplitude Manipulation of Jaw**	TMJ articular and peri-articular structures.	joint-focused manipulative techniques distinct from spinal or soft-tissue approaches.	High-velocity thrusts of Jaw	+ Myofascial release	Osteopath	Department of Dental Sciences	University of Palermo, Italy	Cuccia 2010 [24]
					Physiotherapist	Department of Physical Medicine and Rehabilitation	University of Vienna, Austria	Gesslbauer 2018 [31]
					Chiropractor	Center for Chiropractic Research	University of Iowa, USA	DeVocht 2013 [29]
**High-Velocity Low-Amplitude Manipulation of Upper Dorsal Spine**	Upper thoracic spine	Indirect spinal interventions with hypothesized regional or neurophysiological effects.	High-velocity thrusts applied to upper thoracic spinal segments		Physiotherapist	Program in Physiotherapy	University, São Paulo, Brazil	Packer 2014 [37]
**Massage Therapy**	Masticatory and cervical muscles.	Non-thrust, nonspecific soft-tissue techniques aimed primarily at muscle relaxation.	Sliding and kneading maneuvers on the masseter and temporal muscles. Sliding consisted of a unidirectional movement with constant pressure. Kneading consisted of a gripping maneuver, with movements of compression and decompression.		Physiotherapist	Program in Rehabilitation Sciences	University, São Paulo, Brazil.	Gomes 2014 [32]
**Combination of more techniques**	TMJ, masticatory muscles, cervical spine	Integrated interventions reflecting real-world clinical practice but limiting direct comparability.	Multimodal manual therapy programs combining joint manipulation, soft-tissue techniques, and/or therapeutic exercises. i.e. Jog-manipulation a) closing type with fulcrums on both sides, b) side-to-side type; c) opening type; d) closing type with fulcrums on the impaired side		Dentist	Temporomandibular Disorders and Bruxism Clinic	University of Niigata, Japan	Nagata 2018 [36]

TMJ temporo-mandibular joint.

### 2.4. Data analysis and outcome measures

A meta-analysis was planned a priori and would have been conducted if a sufficient number of studies met predefined methodological criteria. These criteria included: (1) clinical homogeneity of the intervention (similar manual therapy techniques applied to comparable anatomical targets); (2) comparable control conditions (e.g. sham, no treatment, or similar conservative interventions); (3) consistency in outcome measures, specifically pain intensity assessed using validated scales (VAS or NRS) and maximum mouth opening (MMO) measured in millimeters; and (4) comparable follow-up durations allowing pooling of short-term or long-term effects. Due to substantial heterogeneity across included studies in terms of intervention type, treatment dosage, comparator interventions, outcome reporting, and follow-up timing, these criteria were not met. Therefore, quantitative pooling was deemed inappropriate, and a systematic narrative synthesis was conducted in accordance with PRISMA guidelines.

The primary outcomes included pain intensity, assessed using validated scales such as the Visual Analogue Scale (VAS) or Numeric Rating Scale (NRS), and MMO, measured in millimeters using standardized procedures. NRS and VAS were treated as equivalent measures of pain, ranging from 0 (no pain) to 10 or 100 (worst imaginable pain). To standardize these scales, all NRS scores were converted to a 0–10 format to align with the VAS value, allowing for consistent comparisons across studies. Secondary outcomes, when reported, included functional improvement, patient satisfaction, and adverse events.

### 2.5. Risk of bias and quality assessment

**Figure 2.**
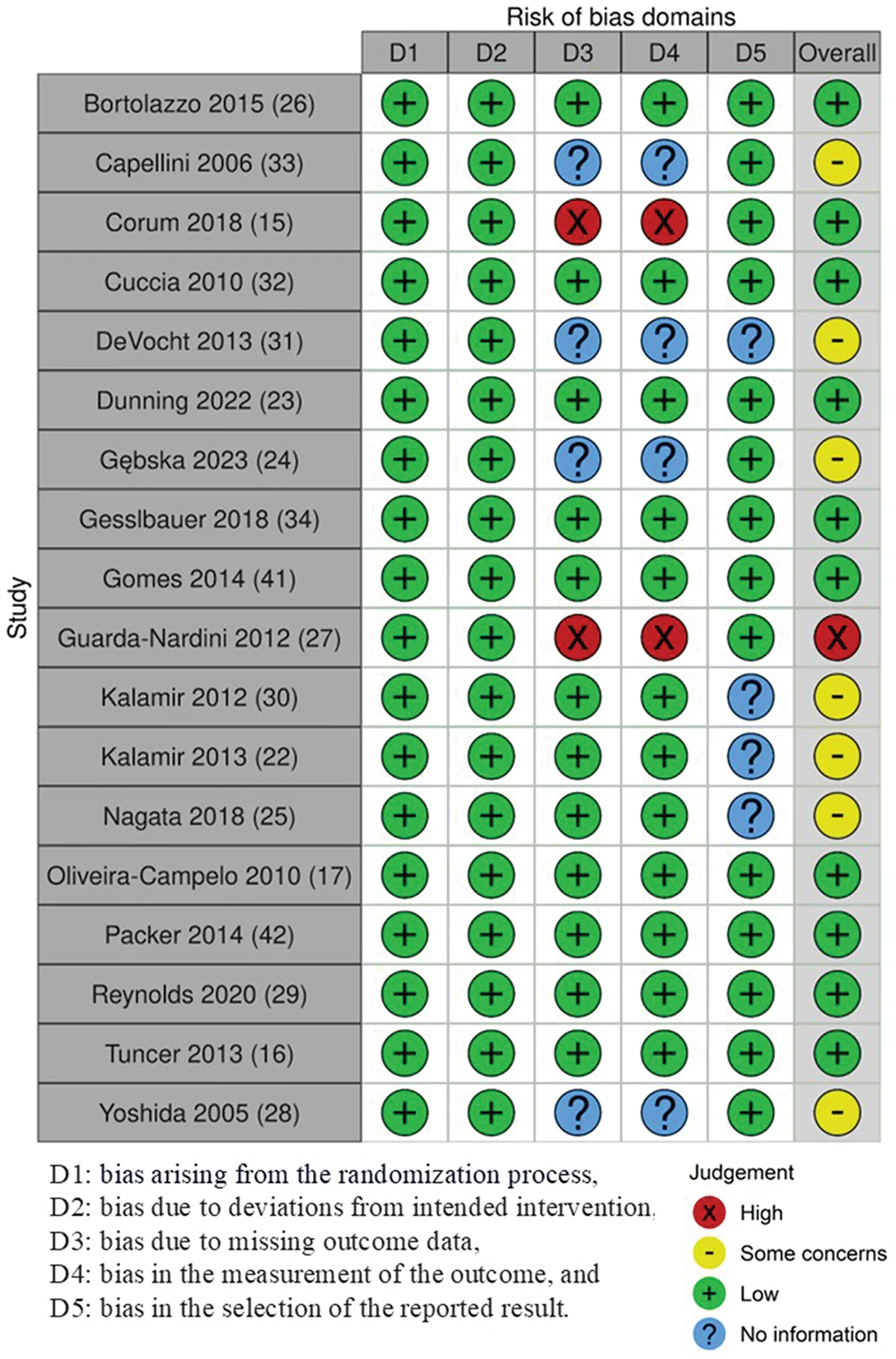
Risk of bias domains of the included RCTs. The Figure 2 provides an overview of the riskofbias assessment for all included RCTs across the RoB 2 domains. Most studies show a low risk of bias in key areas such as randomization and outcome measurement. However, several trials present some concerns, particularly related to missing outcome data and deviations from the intended interventions. A few studies are rated as high risk, reflecting methodological weaknesses that may affect the reliability of their findings. Overall, the table highlights a heterogeneous methodological quality, which should be considered when interpreting the evidence.

Risk of bias was independently assessed by two authors using the Cochrane Risk of Bias tool [39]. Domains evaluated included random sequence generation, allocation concealment, blinding of participants and personnel, incomplete outcome data, selective reporting, and other potential sources of bias. Risk of bias domains of the included RCTs are presented in Figure 2.

The overall quality of evidence was graded using the *Grading of Recommendations, Assessment, Development, and Evaluations* (GRADE) approach, considering study limitations, inconsistency, indirectness, imprecision and publication bias [40–44].

## 3. Results

### 3.1. Description of the studies

A total of 561 articles (85 from PubMed, 365 from Scopus, 44 from Web of Science, 67 from Cochrane Library) were identified. After screening 169 title and abstract, 18 articles met the inclusion criteria and were selected for this systematic review, in accordance with the PICOS framework and as illustrated in the PRISMA 2020 flow diagram (Figure 1).

Primary outcomes included pain intensity and MMO. Secondary outcomes were analyzed across the following subgroups: 1) types of manual therapy (HVLA, myofascial therapy, osteopathic manipulative therapy, other manual techniques); 2) professional categories administering the therapy (physiotherapists, chiropractors, osteopaths, dental specialists and medical doctors); 3) TMD subtypes (myogenous disorders, disc displacements, arthralgia, arthritis, and arthrosis); 4) treatment strategies and professional roles by country (Table 1).

### 3.2. Variations of experimental conditions across the studies

The included RCTs showed substantial heterogeneity in experimental conditions, including manual therapy technique, treatment dosage, follow-up duration, and comparator interventions. For instance, intervention formats ranged from single-sessions treatments (e.g. one atlanto-occipital thrust in Oliveira-Campelo et al. [26]) to multi-week protocols (e.g. eight sessions of dry-needling combined with upper cervical HVLA over four weeks in Dunning et al. [25]). Comparator groups varied widely, including sham or no-treatment control [23,27], standard care [21], splint therapy [25,29,32] and pharmacological treatments [38]. Treatment targets also differed, with some studies focusing solely on masticatory muscles [24,28] while others including cervical or upper-cervical spine structures [21]. These methodological differences underscore the heterogeneity of manual therapy approaches, supporting the decision to rely on a narrative synthesis.

### 3.3. Certainty of evidence for the outcomes

The interpretation of the outcomes was informed by both risk-of-bias assessments and GRADE evaluation. Overall, the certainty of evidence for pain reduction was judged as low to moderate, while the certainty for improvements in maximum mouth opening (MMO) was low.

Downgrading of evidence certainty was primarily driven by methodological limitations, including unclear or high risk of bias in blinding of participants and personnel, incomplete outcome data, and selective reporting in several trials. Additional downgrading factors included clinical heterogeneity in intervention protocols and comparators, as well as imprecision due to small sample sizes and short follow-up periods.

Consequently, although most studies reported short-term improvements in pain and MMO following manual therapy, the overall confidence in these effects remains limited, particularly for long-term outcomes.

### 3.4. Comparative analysis of primary outcomes across studies

#### What specific manual therapy techniques showed significant results?

Across the selected RCTs, manual therapy consistently reduced pain intensity and improved MMO in patients with TMD. Several studies reported statistically improvements when manual therapy was compared to control or alternative interventions.

Fascial Manipulations and massage, administered by physiotherapists, demonstrated efficacy in reducing myogenic TMD symptoms [28,30,32,33]. Myofascial release, performed by physiotherapists, chiropractors and dental nurse, showed significant improvements in both pain and function [22,34,35]. Intra-oral manipulations, particularly thumb pressure techniques, were effective for closed-lock conditions, yielding notable clinical improvements [38]. HVLA techniques applied to the jaw [24,29,31] and cervical spine [21,23,25–27] demonstrated efficacy in reducing pain and improving MMO. HVLA applied to the dorsal spine showed less consistent results [37].

Jaw HVLA combined with myofascial release [24,31], yielded significant pain relief and increased MMO, both immediately after treatment and at 2 months follow-up [24].

### 3.5. Comparative analysis of secondary outcomes

#### 3.5.1. Does the effectiveness of manual therapy for TMD vary by professional practice?

Manual therapy was effective across all professional categories, with significant reductions in pain [21–25] and improvements in MMO [21–26]. However, most studies focused on physiotherapy interventions, often conducted in rehabilitation settings, outnumbering those involving osteopaths, chiropractors, or dental specialists and medical doctors. Physiotherapists consistently achieved significant outcomes in pain reductions [21–23] and MMO improvement [21,22,26]. Osteopathic interventions [24] and physician-led treatments [21] were effective when compared to education- or sham- controls. Chiropractic interventions [29,34] and dental practice-based treatments, including those by dentists [36] and dental nurses [34], also showed positive effects. Studies highlighted the involvement of departments of dental, prosthetic, stomatology sciences [24,30,36] and oral and maxillofacial surgery [33,38].

Across the included randomized controlled trials, manual therapy was consistently associated with reductions in pain intensity and improvements in maximum mouth opening (MMO) in patients with TMD. However, the magnitude and durability of these effects varied considerably across studies. Most trials assessed outcomes over short follow-up periods, ranging from immediate post-treatment to a few months, with limited data available on long-term effectiveness. In addition, substantial heterogeneity was observed in intervention protocols, comparator treatments, outcome measures, and study populations. Several studies were characterized by small sample sizes and limited statistical power. Therefore, while a consistent pattern of short-term positive effects emerged across different manual therapy approaches and professional settings, the certainty of evidence supporting sustained or long-term effectiveness remains limited.

The strongest evidence base emerged from physiotherapy-led or rehabilitation-based practices, likely reflecting their greater representation in the current literature.

The analyses stratified by professional category were conducted to describe how manual therapy interventions for TMD have been investigated across different clinical and research contexts. These comparisons were not intended to imply causal relationships or intrinsic superiority of one professional group over another.

In many included trials, professional background was inherently linked to other study characteristics, such as treatment setting, choice of manual therapy techniques, intervention dosage, comparator interventions, and patient populations. Consequently, observed differences in outcomes across professional categories may reflect study design features and publication patterns rather than profession-specific effectiveness.

#### 3.5.2. Are certain TMD subtypes more responsive to manual therapy?

The most commonly used techniques were HVLA manipulations, accounting for 56% of interventions of the examined literature, and fascial manipulations combined with myofascial release, representing 37.5% of the approaches. These treatments were applied across various professional categories, demonstrating versatility and clinical effectiveness. Approximately 40% of studies employed manipulative physiotherapy, while only 13% involved chiropractors and osteopaths (Table 3).

Key findings by TMD subtype were a) Myofascial TMD: treated with fascial manipulation [28,30,33], myofascial release [34,35], jaw HVLA [29] by chiropractors [29,35], physiotherapists [28,30,33], dental nurses [34]; b) Upper cervical HVLA by physiotherapists [25,27] and medical doctors [21], combined manipulations by dentists [36] and myofascial release by physiotherapists [22]. c) Disc displacement without reduction (closed locking): treated with upper cervical HVLA [23], intra-oral manipulations [38] in physical therapy [23] and oral surgery setting [38]; d) Non-specified TMD: treated by physiotherapists [31] and osteopaths [24] using jaw and upper cervical HVLA [26].

All strategies demonstrated consistent improvements, particularly those involving physiotherapy-based manipulations, which supported both pain reduction and functional recovery.

#### 3.5.3. Which professions are most involved, based on nationality, in the treatment of TMD according to the affiliation of authors in the reported clinical studies?

Physiotherapists are frequently involved in manual therapy for TMD, with contributions from Brazil, Turkey, Europe (Poland, Italy, Spain, Greece and Austria). Chiropractors are more represented in Australia, England, and the USA. Osteopaths play a crucial role in Brazil, Spain and Italy. Medical doctors conducting manual therapy interventions are noted in Turkey. Dentists and dental specialists are involved in intra-oral manipulations and jaw-related interventions, with studies from Japan, Australia and England. Additionally, HVLA of cervical spine [27] and of the jaw combined with myofascial release [24] have been studied in Brazil (Table 3).

Studies on fascial manipulation and myofascial release often originate from physiotherapy and rehabilitation departments in Universities across Brazil, Poland, Turkey, and the USA. Chiropractic-based manipulation and myofascial interventions are mostly conducted in Australia and England, affiliated with chiropractic departments (Table 3).

HVLA are performed across Turkey, Spain, Greece at the upper cervical spine, in Italy and Austria at the jaw, in Brazil at the cervical and dorsal spine, with researchers affiliated with physical therapy, osteopathic, and dental science departments. In USA, HVLA are executed in physical therapy and chiropractic departments at the upper cervical spine and jaw. Dental and maxillofacial interventions are prevalent in Japan, conducted within oral and maxillofacial surgery clinics (Table 3).

Brazil showed strong representation in physiotherapy-based manual therapy, while Australia, USA, and England contributed significantly to chiropractic approaches. Italy, Spain, Greece, Turkey and the USA demonstrated a mixed profile, with both osteopathic and physiotherapy-led interventions across multiple institutions.

## 4. Discussion

### 4.1. Overall interpretation of manual therapy effects in TMD

This systematic review of RCTs examined the role of manual therapy interventions delivered by various healthcare professionals – including physiotherapists, chiropractors, osteopaths, and physicians – in the management of TMD. Manual therapy was defined as a set of hands-on techniques aimed at reducing pain, improving joint mobility, and addressing musculoskeletal dysfunctions associated with TMD [12].

To the best of our knowledge, this is the first systematic review to evaluate the efficacy of manual therapy for TMD across multiple professional categories, offering a comparative perspective on clinical outcomes based on practitioner background. The inclusion of country-based analysis provided contextual insight into the geographical distribution of clinical practices and professional involvement, helping to identify potential variations in therapeutic approaches related to cultural, educational, and healthcare system differences. Nonetheless, the primary objective remained the comparative effectiveness of the manual therapy techniques and the professionals administering them.

### 4.2. Clinical meaning of short-term improvements and durability of effects

According to the findings, manual therapy was generally effective in treating TMD, regardless of the professional performing the intervention. Given the short intervention durations in most studies, with assessments conducted immediately post-treatment, the results suggest that manual therapy can lead to rapid and clinically meaningful pain reductions. The studies by Oliveira-Campelo et al. [26] and Gesslbauer et al. [31] reported immediate symptom relief and increased mandibular range of motion immediately after treatment. Similarly, Dunning et al. [25] and Kalamir et al. [35] observed improvements within six weeks, confirming the short-term efficacy of manual therapy. Longer follow-up periods, ranging from several months to one year, offered insights into the sustainability of therapeutic benefits. Cuccia et al. [24] and DeVocht et al. [29] demonstrated significant improvements at six months, while Kalamir et al. [34] reported sustained efficacy up to one year, particularly in chronic myogenous TMD. These findings suggest that specific techniques, such as chiropractic intraoral manipulation, may yield durable outcomes. The distinction between short- and long-term effects underscores the importance of tailoring manual therapy strategies to the clinical context: short-term interventions may be more suitable for acute symptom relief, whereas long-term approaches are more appropriated for chronic conditions requiring sustained functional recovery.

### 4.3. Technique-specific considerations across TMD subtypes

The evidence indicates a growing trend toward manual therapy as the primary intervention across TMD subtypes, with treatment increasingly tailored to the underlying pathology. Most studies employed the DC/TMD diagnostic criteria [3,45], and the Wilkes TMJ staging system [46]. DC/TMD provides a structured two-axis framework [3,45], Axis I focuses on physical diagnoses (e.g. myofascial pain, disc displacements, and TMJ arthralgia) [47], while Axis II addresses the psychosocial factors and pain-related disability. In this systematic review, most studies applied Axis I criteria [22,25,27,30,35], while a subset incorporated both Axis I and II [21,33] to identify TMD subtypes most responsive to manual therapy. Tailored approaches were emphasized for myogenic, myofascial, and disc displacement subtypes, recognizing that each requires distinct therapeutic strategies based on pathophysiology and clinical presentation. For Axis I TMD, positive outcomes were reported with upper cervical HVLA techniques, likely due to interruption of the nociceptive signaling via trigemino-cervical convergence mechanisms [21,25,27]. Similarly, myofascial release targeting masticatory and jaw muscles showed consistent efficacy [22]. Evidence also supports combining techniques to enhance outcomes. Dunning et al. [25] demonstrated that combining dry needling combined with upper cervical manipulation outperformed splint therapy, diclofenac, and TMJ mobilization. Axis I subtypes, particularly myofascial TMD, responded well to manual therapy interventions [28–30,33–35].

### 4.4. Interpretation of technique-related effects and mechanistic considerations

Myofascial manipulations [28,30,33] and trigger point releases [34,35] were particularly effective in reducing subjective pain perception. However, botulinum toxin injections, as reported by Guarda-Nardini et al. [33], showed slightly greater improvements in jaw range of motion. Intra-oral myofascial therapy also demonstrated pain relief and significant myorelaxant effects [30,35]. Medical doctors and oral health professionals achieved positive outcomes using HVLA, myofascial release, and intraoral manipulation techniques [21,34,36].

### 4.5. Confidence in the evidence and implications for interpretation

Although the majority of included RCTs reported positive effects of manual therapy on pain and function, these findings must be interpreted in light of the identified risk-of-bias concerns and GRADE assessments. Several studies lacked adequate blinding, used small samples, or reported outcomes over short follow-up periods, which limits confidence in the stability and generalizability of the observed effects.

According to GRADE criteria, evidence for pain reduction was downgraded mainly due to risk of bias and imprecision, while evidence for MMO improvement was further downgraded for inconsistency across studies and indirectness related to heterogeneous intervention protocols. As a result, the current evidence supports short-term symptomatic improvements, but provides limited certainty regarding sustained or long-term effectiveness of manual therapy for TMD.

The Wilkes classification system, which categorizes TMJ disorders, from mild disc displacement to advanced degenerative stages, was applied in studies addressing disc displacement cases [46]. For example, Yoshida et al. [38] used intraoral manipulation to treat TMJ internal derangements with ‘closed locking,’ demonstrating clinical efficacy.

### 4.6. Professional background and contextual interpretation of outcomes

While manual therapy appeared broadly effective, its success varied depending on the professional practice. The analysis revealed differences in outcomes across professional categories, likely reflecting variations in technique, training, and therapeutic methodology. These findings underscore the importance of aligning treatment approaches with practitioner expertise and patient-specific needs. Variability in outcomes may reflect the therapist’s level of training, precision and clinical competence [13,14]. Several studies [21,22,25,26,29,31,34] emphasized that manual therapy was performed by professionals with specialized training, reinforcing the notion that clinical expertise is critical to achieving effective results. The success of manual therapy interventions may therefore be contingent on the practitioner’s qualifications and experience [13,14].

In conclusion, the consistent positive outcomes across techniques suggest that manual therapy is broadly effective for TMD, with variations in success linked to TMD subtype and therapeutic modality.

The apparent differences in outcomes reported across professional categories should be interpreted with caution. The professional identity of the clinician was frequently confounded with contextual variables, including clinical setting, therapeutic approach, training background, and regional practice patterns. As such, these findings do not support causal inferences regarding the superiority of any single profession. Rather, the observed distribution of positive outcomes likely reflects how manual therapy for TMD has been studied and reported within different professional communities. The overrepresentation of certain professions, particularly physiotherapists, may be influenced by publication volume and research infrastructure rather than by differences in clinical effectiveness.

### 4.7. Implications for clinical practice and limitations of the study

Although the majority of included studies reported favorable outcomes for manual therapy in the management of TMD, these findings should be interpreted with caution. According to the GRADE framework, the overall certainty of evidence ranged from low to moderate, primarily due to study heterogeneity, risk of bias, imprecision related to small sample sizes, and short follow-up durations. Importantly, the consistency of positive findings across studies does not necessarily equate to high-certainty evidence. While manual therapy demonstrated reproducible short-term benefits in pain reduction and functional improvement, robust conclusions regarding long-term effectiveness cannot be drawn based on the current evidence base.

Due to the limited number of studies and small sample sizes, a quantitative meta-analysis was not feasible. As a results, the conclusions may be subject to limitations. Future RCTs with larger cohorts and standardized methodologies are needed to strengthen the evidence base. The included studies exhibited significant heterogeneity in terms of TMD diagnosis (e.g. myogenic, myofascial, disc displacement), severity and chronicity, types of manual therapy applied (e.g. HVLA, myofascial release), professionals background of the therapist, and intervention timing and duration. Variability in follow-up periods and outcome measures further complicated cross-study comparisons. Moreover, most studies assessed outcomes immediately post-treatment, with limited data on long-term efficacy, hindering conclusions about the durability of manual therapy effects. Additionally, the terminology used to describe professional categories was derived directly from the cited articles, based on the qualifications and roles assigned by the original authors. Finally, although the search strategy was restricted to English-language publications, the screening process did not identify relevant RCTs excluded on the basis of language alone. Nevertheless, the possibility that some non-English evidence exists cannot be entirely excluded, and this should be considered when interpreting the international scope of the findings.

## 5. Conclusions

Manual therapy represents a valuable option in the management of TMD, highlighting the interdisciplinary nature of treatment and demonstrating clinical effectiveness regardless of the specific technique or professional category involved.

The findings of this systematic review of RCTs provide key insights into the application of manual therapy for TMD, addressing several critical aspects: 1) Efficacy in reducing pain and improving functional outcomes, consistently observed across studies and independent of the professional performing the intervention; 2) Predominant focus on Axis I of the DC/TMD diagnostic criteria, encompassing muscle disorders and disc displacements, with positive results reported for techniques such as upper cervical spine HVLA and myofascial release; 3) Broad effectiveness of manual therapy across countries and professional practices, with physiotherapists leading the majority of interventions, likely due to the greater volume of published evidence. These findings should be interpreted considering the low-to-moderate certainty of evidence, with most effects supported by short-term data and limited by methodological constraints.

This systematic review suggests that manual therapy is associated with short-term improvements in pain and mandibular function in patients with temporomandibular disorders, as measured by patient-reported pain intensity and maximum mouth opening. Positive effects were observed across different manual therapy techniques and professional settings. However, the available evidence is predominantly short-term and heterogeneous, with several studies being underpowered. Therefore, while the findings indicate a consistent trend toward short-term benefit, the strength and certainty of evidence supporting long-term effectiveness and equivalence across professional categories remain limited. Future well-designed, adequately powered randomized controlled trials with longer follow-up periods are needed to confirm these findings. Comparisons across professional categories should be regarded as descriptive and hypothesis-generating, as they are influenced by study design characteristics and publication patterns rather than isolated professional effects

Moreover, most RCTs emphasized the importance of accurate diagnosis, specialized training, and clinical expertise. These findings underscore the need to develop tailored treatment protocols and reinforce the value of an interdisciplinary approach to TMD management. Manual therapy should be administered exclusively by authorized and licensed professionals with appropriate training, ensuring safety and therapeutic efficacy. Moreover, the high variability in TMD subtypes, treatment settings, professional background, and manual techniques highlights the need for more standardized and methodologically robust studies. Future research should prioritize the development of consistent intervention protocols, extended follow-up durations, and comprehensive outcomes reporting, in orders to strengthen the reliability and applicability of findings and support the integration of manual therapy into evidence-based clinical practice.
